# The Application of Gas Dwell Time Control for Rapid Single Wall Carbon Nanotube Forest Synthesis to Acetylene Feedstock

**DOI:** 10.3390/nano5031200

**Published:** 2015-07-17

**Authors:** Naoyuki Matsumoto, Azusa Oshima, Shunsuke Sakurai, Takeo Yamada, Motoo Yumura, Kenji Hata, Don N. Futaba

**Affiliations:** National Institute of Advanced Industrial Science and Technology (AIST), Central 5, 1-1-1 Higashi, Tsukuba, Ibaraki 305-8565, Japan; E-Mails: matsumoto-naoyuki@aist.go.jp (N.M.); oshima-azusa@aist.go.jp (A.O.); shunsuke-sakurai@aist.go.jp (S.S.); takeo-yamada@aist.go.jp (T.Y.); m.yumura@aist.go.jp (M.Y.)

**Keywords:** single-walled carbon nanotubes, water-assisted chemical vapor deposition, carbon flux, dwell time, acetylene

## Abstract

One aspect of carbon nanotube (CNT) synthesis that remains an obstacle to realize industrial mass production is the growth efficiency. Many approaches have been reported to improve the efficiency, either by lengthening the catalyst lifetime or by increasing the growth rate. We investigated the applicability of dwell time and carbon flux control to optimize yield, growth rate, and catalyst lifetime of water-assisted chemical vapor deposition of single-walled carbon nanotube (SWCNT) forests using acetylene as a carbon feedstock. Our results show that although acetylene is a precursor to CNT synthesis and possesses a high reactivity, the SWCNT forest growth efficiency is highly sensitive to dwell time and carbon flux similar to ethylene. Through a systematic study spanning a wide range of dwell time and carbon flux levels, the relationship of the height, growth rate, and catalyst lifetime is found. Further, for the optimum conditions for 10 min growth, SWCNT forests with ~2500 μm height, ~350 μm/min initial growth rates and extended lifetimes could be achieved by increasing the dwell time to ~5 s, demonstrating the generality of dwell time control to highly reactive gases.

## 1. Introduction

One of the most significant obstacles in limiting the application of single-walled carbon nanotubes (SWCNTs) is the low growth efficiency. Immense research has been conducted over the past 20 years to address this aspect to improve the carbon nanotube (CNT) growth process. Apt examples include varied hydrocarbon carbon feedstock, the use of additive gases (water, CO_2_, *etc.*), plasmas, hot filament, catalyst buffer materials, porous substrates, alloyed catalysts, and a variety of conditions and ambient [1,2,3,4,5,6,7,8,9,10,11,12,13,14,15]. Consequently, CNT synthesis technology has advanced to a stage where millimeter-scale tall, vertically aligned SWCNTs arrays, or “forests”, can be routinely synthesized for large-scale production. Further, the SWCNTs within these forests have shown to possess exceptional properties, such as high purity, alignment, high surface area, and long length [7,8,16,17,18]. These properties have afforded the development of CNT applications, exemplified by strain sensors, aerogel muscles, electro-catalysts for fuel cells, stretchable conductors, super-capacitors, microfluidic chips, electric motors and generators, heat exchangers as thermal/electrical conductive polymers (rubber), and metal and ceramic composites [19,20,21,22,23,24,25,26,27,28].

Examination of previous reports has shown that the methods of highly efficient growth of SWCNTs fall into two general groups. In one group, SWCNT forest growth is characterized by long catalyst lifetimes and slow SWCNT growth rates. For example, the point-arc microwave plasma chemical vapor deposition (CVD) method has demonstrated the synthesis of 5-mm tall SWCNT forests (~2.6 μm/min growth rate with a ~32 h growth time) [29]. In addition, the alcohol CVD method has demonstrated a growth rate of a few μm/min, which exponentially decreased and terminated after ~30 min [30]. Furthermore, using a rapid heating cold-wall low-pressure synthesis method, SWCNT forests have been shown to grown to millimeter-scale heights over a period of 60 min [31]. Although this group exhibits long catalyst lifetimes, the growth rates are slow, and thus the resultant production rates are not high. In contrast, the second group is characterized by fast growth rates but short lifetimes. For example, the water-assisted CVD method exhibited a growth rate of ~300 μm/min for SWCNT forests, and the growth rate terminated within 20 min at the height of ~970 mm [32]. Further, Maruyama *et al.* have reported an acetylene-accelerated alcohol CVD with enhanced growth rate (initial growth rate of ~100 mm/min), yet the lifetime was short (a few min) [33]. Noda *et al.* have demonstrated millimeter-scale SWCNT arrays using an acetylene carbon feedstock within a 10-min growth time yet with a short catalyst lifetime (~15 min) [34]. This group is characterized by the use of a highly reactive carbon feedstock or a carbon feedstock with an additive gas (water or alcohol). The need of the additive gas has been proposed to support the catalyst resilience to bear rapid growth.

One approach has been proposed to bridge the two categories by achieving both rapid growth and long lifetime [35]. In this approach (denoted “Fast-CVD”), using an ethylene carbon feedstock, the gas dwell time (gas heating time prior to impingement to the catalysts), which is usually fixed by the dimension of the furnace, was treated as a variable parameter by adjusting the separation between gas input and sample stage. The growth yield (height) was found to significantly depend on the dwell time, and exceptionally high growth rates (~620 μm/min) were demonstrated when the dwell time was extended from 4 to 7 s. Therefore, this approach demonstrated its promise to achieve highly efficient growth with high growth rate and long lifetime; however, its feasibility toward other carbon feedstocks remained unclear.

In this research, we investigated the applicability of the “Fast-CVD” approach for high efficiency SWCNT synthesis to an acetylene carbon feedstock. Acetylene is a commonly used carbon feedstock known for its high reactivity and the ability to grow forests without the use of a growth enhancer [34,36,37]. Our results showed that the yield, growth rate (measured as the height), and catalyst lifetime could be all improved and optimized in a similar manner as ethylene. In addition, SWCNT yield (height) as high as 2500 μm and initial growth rates as high as 350 μm/min for a 10-min growth could be achieved as well as extending the catalyst lifetimes. Furthermore, in comparison to the results of Fast-CVD approach with ethylene, the optimum carbon flux occurred at ~25-times lower level, the optimum dwell time was shorter (~5 s for acetylene *vs.* ~7s for ethylene), and the corresponding carbon efficiency was greater by more than 15-times higher (32%). Altogether, acetylene-based water-assisted CVD method toward an optimum growth of SWCNT forests is established.

## 2. Results and Discussion

**Figure 1:**
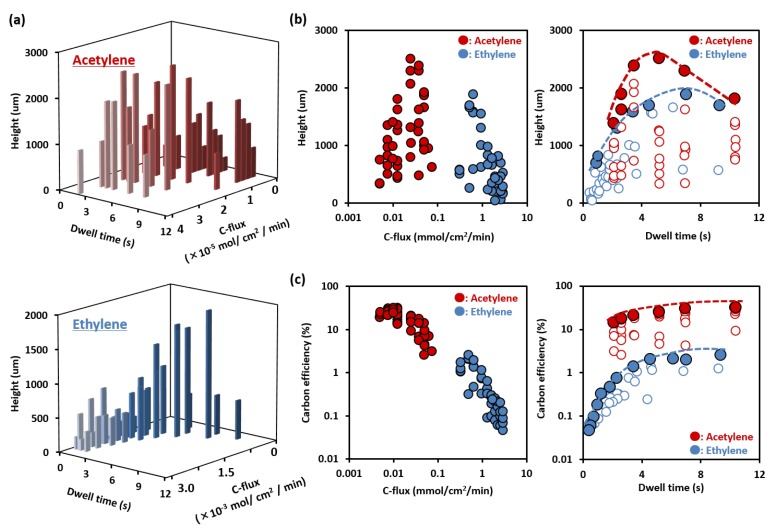
(**a**) Three-dimensional mapping of single-walled carbon nanotubes (SWCNTs) height as functions of dwell time and carbon flux for acetylene (upper), ethylene (lower); (**b**) Two-dimensional mapping of SWCNT height as a function of dwell time and carbon flux. Peak positions at each dwell time are marked as solid circles; (**c**) Carbon efficiency as a function of the dwell time and carbon flux. Peak positions at each dwell time are marked as solid circles.

Growth characterization revealed the sensitivity of the acetylene-grown SWCNTs to the dwell time and carbon flux. As a function of the dwell time and carbon flux, the SWCNT forests grown for 10 min from acetylene and ethylene were characterized by the forest height (Figure 1a,b). We note that the 2D plots of the height as functions of C-flux and dwell-time are projections of the height data from the 3D plots onto their respective axes. The optimum data is indicated as solid dots, from which the trends are discussed. First, and central to our work, a significant increase in the CNT height for the acetylene-based growth was observed with increasing dwell time that peaked at ~5 s. The maximum height (~2500 μm) represents a ~2.9 times improvement compared to standard super-growth SWCNTs synthesis using ethylene (860 μm) [35]. This means that the Fast-CVD approach is not strictly limited to an ethylene ambient. Transmission electron microscopy (TEM) observations confirmed that the grown CNTs were SWCNTs (average wall number: ~1.1) (Figure S1 in Supplemental Materials). In addition, outer-specific surface areas were estimated from nitrogen adsorption isotherms as 1111 and 1130 m^2^/g for acetylene and ethylene, respectively, which is identical with that of SWCNTs grown under standard growth conditions [38], indicating both high SWCNT selectivity and purity. Note: the outer-specific surface area for a perfectly clean and closed SWCNT is 1315 m^2^/g [38,39]. The measured outer-surface area of the SWCNT forests corresponds to ~95% absolute purity, *i.e.*, the carbon nanotube weight percent of a CNT sample. This means that the amount of carbonaceous impurities was ~5%. Scanning electron microscope (SEM) images show that the forest is not heavily bundled as to limit surface area analysis, and TEM analysis shows the presence of small amounts of carbon impurities adsorbed onto the tubes, which is in agreement with the surface area measurements (Figure S1). These results show that despite the extended dwell time, and the carbon feedstock, particularly acetylene, did not suffer from excessive gas-phase decomposition that would result in soot formation, an increase in carbonaceous impurities and the corresponding decrease in specific surface area (SSA): it was not the case as evidenced by the outer-SSA of ~837 m^2^/g for a dwell time of 10.3 s. This observed decrease in SSA is in agreement with reported dependence of SWCNT SSA and the amount of carbonaceous impurities [38]. The carbon flux was optimized along with the dwell time. Similar to the results observed with ethylene, the achieved forest height increased despite a decrease in the carbon source, indicating improvement in the conversion rate efficiency of the carbon source gas into CNTs for this highly reactive carbon feedstock of acetylene (Figure 1a,b). This phenomenon has been previously interpreted that the decreased carbon flux suppressed contamination of the growth environment by soot [39]. These results indicate that the growth yield of water-assisted CVD could be significantly improved by optimizing the dwell time and carbon flux for highly reactive carbon feedstock, such as acetylene, without sacrificing the quality of the SWCNTs.

To investigate the differences between the acetylene and ethylene growth ambient, the SWCNT height and the carbon efficiency were plotted as functions of the carbon flux and dwell time (Figure 1b). From these plots, we made the following observations. First, optimization in height is a result of the optimization of both the C-flux and dwell time. Second, both show a clear peaking in the height. Third, the carbon-flux range and peak-height location for acetylene is ~25 times smaller than that of ethylene (acetylene: 2.4 × 10^−5^ mol/cm^2^/min, ethylene: 6.0 × 10^−4^ mol/cm^2^/min). Fourth, the peak in the yield (height) does not occur at the highest acetylene flux levels. Fifth, the height dependence on the dwell time for both ambients showed gradual increase, peaking, and a slow decline with dwell time, but the peak location for acetylene occurred at ~5 s, which was shorter than ~7 s for ethylene (Figure 1b). These values are in agreement with Youn *et al.* who demonstrated the importance of gas heating for acetylene feedstock in a low-pressure synthesis system [36].

The carbon efficiency for both growth ambients (acetylene and ethylene) decreased with increased carbon flux, but as the input level of ethylene was ~15-times higher, the carbon efficiency is significantly higher for acetylene than for ethylene (Figure 1c). The optimum points are indicated as solid dots and from this, the trends are discussed. We note that the carbon efficiency is calculated as the quotient of the masses of the SWCNT forest and the entire carbon input amount for a three-inch furnace and a 4 cm^2^ substrate, so much of the carbon feedstock never contacted the substrate. This result is in agreement with previous reports, which demonstrated acetylene is a precursor in CNT growth, while ethylene requires additional decomposition into the same form [33,34]. From the plot of carbon efficiency *versus* dwell time, ethylene exhibited a sharp increase from 0 to 2 s followed by a plateauing in the carbon efficiency. In contrast, acetylene showed only a moderate rise in carbon efficiency with dwell time. Therefore, increased carbon conversion efficiency was highest for a combination of increased gas dwell time and reduced C-flux. We believe that the underlying mechanism explaining the selective increase of carbon conversion into CNTs without affecting catalyst deactivation (*i.e.*, lifetime) centers on the reduced levels of carbon feedstock due to appropriate thermal treatment. As observed in our data, optimized yield resulted from increased dwell-time and a reduced C-flux. This indicates that the efficiency of the carbon conversion increased because higher yields were achieved with lower C-flux levels. Further this finding indicates that because the initial amount is reduced, the absolute unused fraction is reduced and contributes less to catalyst deactivation. In addition, the increase in yield with decreased C-flux directly carries over to higher carbon efficiencies as observed.

**Figure 2:**
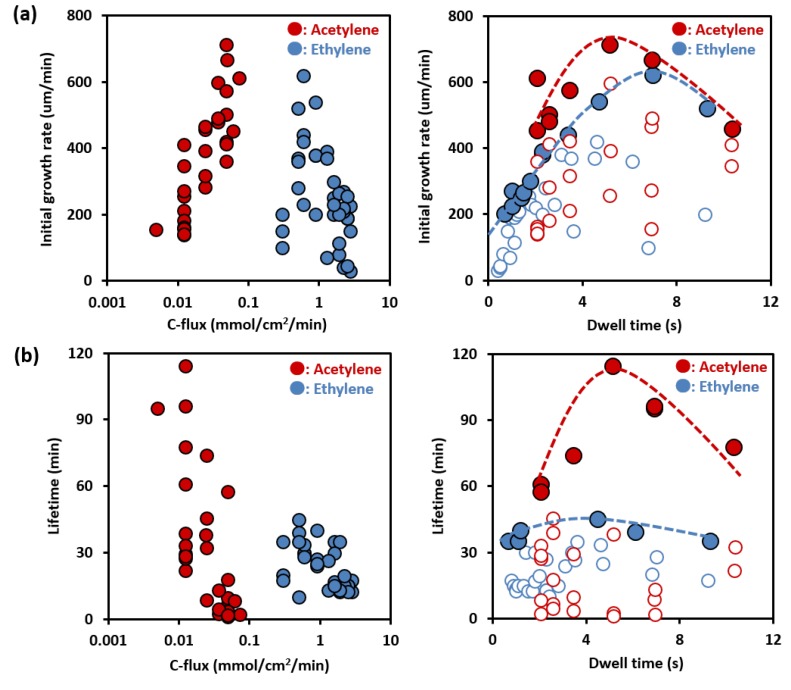
Two-dimensional mapping of (**a**) initial growth rate; and (**b**) life time as functions of dwell time and carbon flux. Blue and red circles show SWCNT growth using ethylene and acetylene, respectively. (Growth temperature: 1083 K of acetylene, 1073 K of ethylene, growth time: 10 min).

To further compare the Fast-CVD effects between acetylene and ethylene growth ambient, we investigated the dependence of the growth dynamics on the dwell time and carbon flux. The time evolution and growth curves for each forest across the diverse range of dwell times and carbon fluxes for both the acetylene and ethylene growth ambients were measured using an *in-situ* telecentric height monitoring system [40]. While the growth conditions spanned a wide range of dwell times and carbon fluxes, all of the growth curves exhibited a similar overall trend, *i.e.*, the growth rate was the highest at the onset of growth, gradually decreased, and finally terminated, as reported previously [32,40]. According to the first order kinetics, the growth curve of the height (yield) of the forest could be described as:

(1) $$H=\beta \tau_{o}(1-e^{-t/\tau }{}^{\circ})$$

where *H* is the forest height and the two fitting parameters, β and τ_*o*_, represent the initial growth rate (IGR) and lifetime, respectively [32,40]. By fitting each growth curve to this growth equation, the IGR and the catalyst lifetime, which characterized the evolution of the growth, could be determined and plotted as functions of dwell time and carbon flux, respectively for both acetylene and ethylene (Figure 2a,b). With increased carbon flux the IGR for the acetylene growth ambient showed a sharp increase, which was nearly identical to that observed for the ethylene growth ambient. Further, with increase in gas dwell time, the IGR peaked at ~715 μm/min at a dwell time of 5 s, and then decreased with increased dwell time. The growth rate of 715 μm/min is a ~2.9-times improvement from previous water-assisted growth (~250 μm/min), and represents a new benchmark for the fast growth rate of SWCNTs [32,35]. It should be noted that the trend indicated in Figure 2b represents the peak height among several carbon flux levels at each dwell time condition. As seen in these figures, this general behavior with carbon fluxes and dwell time was similar to that of ethylene with several significant differences. First, for acetylene, the optimum carbon flux occurs at about 15-times lower level than that observed for ethylene. Second, the optimum dwell time for acetylene occurs at ~5 s compared to the 7 s for ethylene. Third, the optimum lifetime for acetylene is several times longer than that observed for ethylene, indicating that when properly optimized, acetylene is less prone to deactivating the catalyst [31,33,34,36,37]. Fourth, the dependence of catalyst lifetime for acetylene is highly sensitive to dwell time.

These results follow the underlying mechanism describing a highly carbon efficient growth. In short, the basic reaction processes of water-assisted CVD can be described as the following: When carbon feedstock enters the reaction zone, the feedstock either contributes to (1) the *synthesis* of CNTs or (2) the *deactivation* of catalysts by carbon coating. The addition of water acts to removal of carbon coating to maintain catalyst activity [35]. As the synthesis and deactivation are associated with the lifetime and growth rate, respectively, the synthesis of CNTs and catalyst deactivation represent competing pathways. Hence, the result is the commonly observed inverse relationship between the growth rate and lifetime. Our results here show that proper adjustment of the dwell time and carbon flux can invoke a preferential shift toward the synthesis of CNTs. This shift to the synthesis direction occurred because the acetylene feedstock was treated to the optimum thermal history upon arrival to the catalyst site. This would result in the increased growth rate, reduced carbon flux, and increased carbon efficiency, as observed experimentally. Further, insufficient heating would lead to less efficient growth, and excessive heating would lead to a sharp decrease in catalyst lifetime.

We punctuate one point that remains unclear regarding the dependence of the synthesis of SWCNT forests using acetylene on the dwell time and carbon flux. It has been reported that acetylene is a precursor for SWCNTs synthesis [31,33,34,36,37,41,42,43]. However, we cannot explain the strong experimentally observed dependence of the yield (height) on the dwell time (5 s for acetylene *vs.* 7 s for ethylene), where we would have expected the dependence to be much less prominent than the case of ethylene. The need for gas heating to achieve high growth rate is also supported by Youn *et al.* [31] who use a low pressure, cold wall CVD to grow SWCNT forest and demonstrate millimeter scale heights in a 60 min growth time. Therefore, while our results in no way contradict previous reports of acetylene as a precursor for CNT synthesis, our results show that a precise level of energy, through heating, is still required to optimize the conversion of acetylene molecules to CNTs [31,33,34,36,41,42,43]. Further, while the precise condition of the acetylene feedstock remains unknown, the dependence on dwell time gives rise to the possibility that gas phase reactions may play a factor [37,44].

We note that the optimum values for dwell time and carbon flux would likely be dependent on reactor design, such as furnace heat distribution, size, shower design, *etc.*, and therefore our dwell time of 5 s may not be completely general to all synthetic systems. However, the central point that we emphasize is the need to appropriately optimize the gas heating time and carbon flux even for a highly reactive carbon feedstock as acetylene.

## 3. Materials and Methods

The central concept of this work was to apply the Fast-CVD approach, *i.e.*, dwell time and carbon flux tuning, to an acetylene growth ambient in order to control and improve CNT forest growth. Carbon flux was simply defined as the amount of carbon impinging onto the catalysts per unit time per unit area. By this approach, we could maximize the conversion rate of the carbon source to CNTs at the catalyst sites by optimizing the pyrolysis of the carbon source. To regulate the dwell time and carbon flux over a wide range, we constructed a chemical vapor deposition (CVD) reactor composed of a three-inch, infrared-heated vertical furnace with a long heating length equipped with a gas shower head with the face oriented parallel to the substrate and connected to the gas inlet tube [35,45]. This system allowed for the uniform increase in the dwell time of the acetylene feedstock. It should be noted that in contrast to the previous report by Yasuda *et al.*, the dwell time was controlled by modulating the total gas flow (500–5000 sccm) for corresponding acetylene levels (2–30 sccm), while the carbon flux was modulated by adjusting both the total gas flow of the carrier gas and the acetylene concentration (flow rate). By employing the previously reported calculation methodology [35], the dwell time (*t*) and the carbon flux (*Fc*) were calculated as described below:

*t* = 273 (*Ad*/*FT_GT_*) (2)

*Fc* = *f*/(11.2 *A*) (3)

where *A* is the cross sectional area of the shower head, *d* is the length of the gas heating zone, *F* is the total inlet gas flow (500–5000 sccm), and *T_GT_* is the furnace temperature, and *f* is the acetylene flow rate (2–30 sccm), respectively [35]. In this paper, we fixed the heating length (*d* = 15.0 cm), cross sectional area of the shower head (*A* = 44.2 cm^2^), and furnace temperature (*T_GT_* = 1083 K). From Equations (1) and (2), we could calculate the dwell time and carbon flux for each condition SWCNT forests were synthesized on 4 cm^2^ silicon substrates with Al_2_O_3_ (40 nm)/Fe (1.8 nm) catalysts at 1083 K by water-assisted CVD. The catalyst was fixed not to influence the wall number and diameter of the grown CNTs. He, H_2_, acetylene/ethylene, and water were used as carrier gas, catalyst reduction gas, carbon source, and growth enhancer, respectively.

## 4. Conclusions

In conclusion, we have experimentally demonstrated the generality of the dwell-time and carbon-flux control of the carbon feedstock (Fast-CVD) for the acetylene-based SWCNT CVD. Our results showed that the yield (height), initial growth rate, and catalyst lifetime could be optimized. Yield as high as 2500 μm, initial growth rates as high as 700 μm/min, and extended catalyst lifetime could be achieved at each optimum growth conditions. This growth rate represents a new benchmark for SWCNT forest synthesis on substrates. Furthermore, comparison to the results of Fast-CVD approach on ethylene revealed that the optimum carbon flux occurs at one fifteenth, the optimum dwell time is shorter (5 s for acetylene *vs.* 7 s for ethylene), and the corresponding carbon efficiency can be 15-times higher. The applicability of gas-dwell-time and carbon-flux tuning to the water-assisted SWCNT forest synthesis based on acetylene demonstrates an approach of achieving fast SWCNT growth with high growth rate for both fundamental science and industrial mass production.

## Supplementary Materials

Supplementary materials can be accessed at: http://www.mdpi.com/2079-4991/5/3/1200/s1.
