# Supplementary material for: The Application of Gas Dwell Time Control for Rapid Single Wall Carbon Nanotube Forest Synthesis to Acetylene Feedstock
Source: Nanomaterials (Basel). 2015 Jul 17;5(3):1200–10. doi: 10.3390/nano5031200 (PMC5304639; doi:10.3390/nano5031200)
Supplement: Supplementary file 1 [file nanomaterials-05-01200-s001.pdf]

Supplementary Information

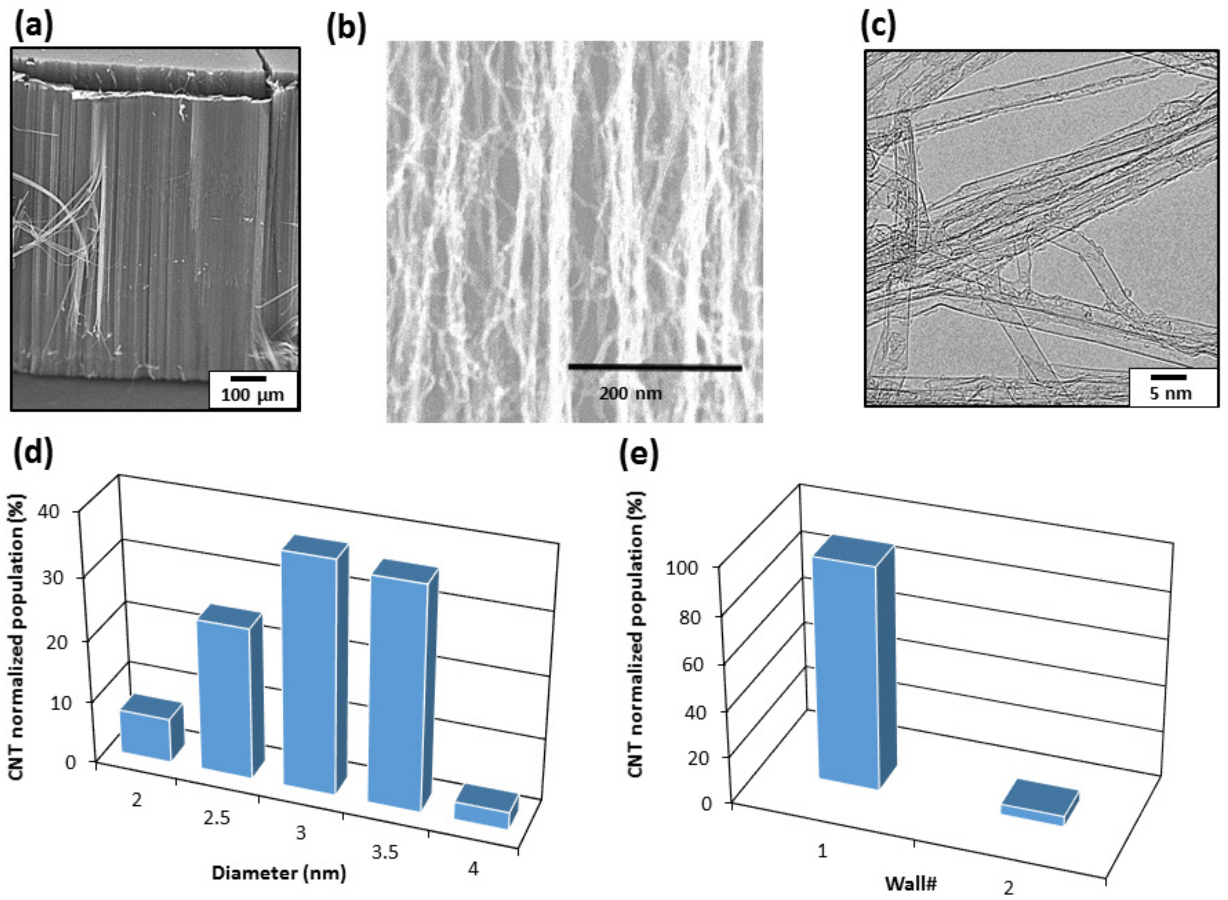

Figure S1. Stucture of carbon nanotube (CNT) synthesized by using acetylene. Scanning electron microscope (SEM) image: (**a**) low and (**b**) high magnification, (**c**) transmission electron microscopy (TEM) image, and histograms of the (**d**) wall number and (**e**) diameter distribution from TEM images.

© 2015 by the author; licensee MDPI, Basel, Switzerland. This article is an open access article distributed under the terms and conditions of the Creative Commons Attribution license (http://creativecommons.org/licenses/by/4.0/).
